# Comparative functional genomic screens of three yeast deletion collections reveal unexpected effects of genotype in response to diverse stress

**DOI:** 10.1098/rsob.160330

**Published:** 2017-06-07

**Authors:** Erica Acton, Amy Huei-Yi Lee, Pei Jun Zhao, Stephane Flibotte, Mauricio Neira, Sunita Sinha, Jennifer Chiang, Patrick Flaherty, Corey Nislow, Guri Giaever

**Affiliations:** 1 Department of Pharmaceutical Sciences, University of British Columbia, Vancouver, British Columbia, Canada; 2 Department of Genome Science and Technology, University of British Columbia, Vancouver, British Columbia, Canada; 3 Department of Microbiology and Immunology, University of British Columbia, Vancouver, British Columbia, Canada; 4 Department of Zoology and Michael Smith Laboratories, University of British Columbia, Vancouver, British Columbia, Canada; 5 Schulich School of Medicine and Dentistry, Western University, London, Ontario, Canada; 6 Department of Mathematics and Statistics, University of Massachusetts, Amherst, MA, USA

**Keywords:** yeast deletion collection, *Saccharomyces cerevisiae*, functional genomics, genome-wide fitness assay, gene–environment correlation, prototrophy

## Abstract

The Yeast Knockout (YKO) collection has provided a wealth of functional annotations from genome-wide screens. An unintended consequence is that 76% of gene annotations derive from one genotype. The nutritional auxotrophies in the YKO, in particular, have phenotypic consequences. To address this issue, ‘prototrophic’ versions of the YKO collection have been constructed, either by introducing a plasmid carrying wild-type copies of the auxotrophic markers (Plasmid-Borne, PB_prot_) or by backcrossing (Backcrossed, BC_prot_) to a wild-type strain. To systematically assess the impact of the auxotrophies, genome-wide fitness profiles of prototrophic and auxotrophic collections were compared across diverse drug and environmental conditions in 250 experiments. Our quantitative profiles uncovered broad impacts of genotype on phenotype for three deletion collections, and revealed genotypic and strain-construction-specific phenotypes. The PB_prot_ collection exhibited fitness defects associated with plasmid maintenance, while BC_prot_ fitness profiles were compromised due to strain loss from nutrient selection steps during strain construction. The repaired prototrophic versions of the YKO collection did not restore wild-type behaviour nor did they clarify gaps in gene annotation resulting from the auxotrophic background. To remove marker bias and expand the experimental scope of deletion libraries, construction of a *bona fide* prototrophic collection from a wild-type strain will be required.

## 1. Background

Yeast has served as a model eukaryote for biological research for over a century. In the ‘pre-sequence’ era (prior to 1996), in order to isolate effects of genotype on phenotype, mutant validation required tedious backcrossing to wild-type. In the genomic era, the combination of a well-annotated genome sequence with new PCR-based gene disruption technologies made reverse genetics straightforward, allowing the construction of the Yeast Knockout (YKO_aux_) collection, the first and only genome-wide set of precise start-to-stop gene deletions comprising approximately 6000 strains representing the yeast genome [1,2]. The YKO_aux_ collection has greatly expanded our understanding of gene function and the cellular response to perturbation through comprehensive screens performed in thousands of different environmental and drug conditions [1,2]. As a result, in the 15 years since the completion of the YKO_aux_ deletion collection, the proportion of the genome with functional annotation has increased from approximately 30% to 90% [3–7].

For historical reasons an auxotrophic derivative of S288c, a *Saccharomyces cerevisiae* wild-type, was chosen as the parent strain of the YKO_aux_ collection. The auxotrophies (table 1) were included to facilitate genetic manipulations and at the time were considered inert. A decade of functional genomic, proteomic and metabolomic studies have revealed that these auxotrophies are far from benign and have clear impacts on cellular physiology. Compared with the prototrophic parent, the YKO_aux_ genotype exhibits slower growth rates, decreased survival in starvation conditions and altered patterns of gene expression [9–15]. In addition to auxotrophic effects, natural variants present in the S288c parent strain manifest genotype-specific traits that include poor sporulation and increased rates of mitochondrial genome loss [16–19]. Furthermore, within the *S. cerevisiae* clade, S288c has been classified as an outlier both at the sequence level and by comparative phenotyping [4,20].

**Table 1.** Genotypes of deletion collections used in this study.

strain	background	genotype	source
YKO_aux_	BY4743	*MATa/α his3Δ1/his3Δ1 leu2Δ0 /leu2Δ0 LYS2/lys2Δ0 met17Δ0/MET17 ura3Δ0 /ura3Δ0*	Giaever *et al*. [2]; Winzeler *et al*. [1].
BC_prot_	BY4741	*MATa can1Δ::STE2pr-SpHIS5 his3Δ1 lyp1Δ0*	VanderSluis *et al*. [8].
PB_prot_	BY4741	*MATa his3Δ1 leu2Δ0 met17Δ0 ura3Δ0* + pHLUM (Addgene ID 40276)	Mulleder *et al*. [9].
*MATa*	BY4741	*MATa his3Δ1 leu2Δ0 met17Δ0 ura3Δ0*	Giaever *et al*. [2]; Winzeler *et al.* [1].

Two prototrophic versions of the YKO *MATa* collection have been constructed to address the potential confounding effects of auxotrophy and, importantly, enable metabolomic studies without nutrient supplementation [21–23]. In the first case, the ‘Plasmid-Borne’ (PB_prot_) prototrophic collection, auxotrophies were genetically complemented by introducing a single-copy ARS-CEN plasmid carrying wild-type copies of the *HIS3*, *LEU2*, *URA3* and *MET17* genes (pHLUM) [9]. In the second ‘Backcrossed’ (BC_prot_) collection, the auxotrophic markers were repaired using a synthetic genetic array (SGA)-based methodology [24] by backcrossing to a strain prototrophic for the auxotrophic markers (*LEU2*, *URA3* and *MET17* from *S. cerevisiae* and *HIS5* from *Schizosaccharomyces pombe*) and carrying deletions in the arginine (*CAN1)* and lysine (*LYP1)* transporters [25] (ACY742:*MATα can1Δ::STE2pr-SpHIS5 his3Δ1 lyp1Δ0*). Prototrophic *MATa* haploid deletion strains capable of growing on minimal media (MM) were then selected (table 1).

A powerful experimental feature of the YKO_aux_ collection is the presence of two unique 20-base-pair sequences linked to each deletion strain that serve as unique strain identifiers. These tags, or barcodes, enable the fitness of each strain to be analysed in parallel by pooling strains in competitive growth assays. The relative abundance of barcodes representing each strain is then quantitatively assessed by microarray signal intensity [26,27] or by counting barcode read-outs by sequencing (Bar-seq) [28–31]. In addition to fitness profiling, barcodes provide protection from inevitable mix-ups in strain inventory. We took advantage of the unbiased metric of the molecular barcodes to systematically characterize in parallel the phenotypic behaviour of the PB_prot_ and BC_prot_ deletion sets with the original YKO_aux_ collection by performing chemogenomic and environmental fitness profiling in diverse drug and media conditions. We chose the YKO_aux_ homozygous deletion collection for our reference collection after confirming equivalent fitness profiles to the *MATa* haploid collection in a number of synthetic dropout conditions using Bar-seq (electronic supplementary material, figure S1). This choice was due to the availability of more than 3000 YKO_aux_ screens produced by our laboratory in that background [32]. Comparative analysis of the resulting fitness profiles reveals both genotypic (biological) and technical effects of the methodology used to restore prototrophy. Here, we highlight the advantages and disadvantages of each collection for the interrogation of specific biological processes, including findings of significant strain loss and the inability to fully restore wild-type behaviour.

## 2. Results

### 2.1. Genetic roster of each deletion collection

**Figure 1.**
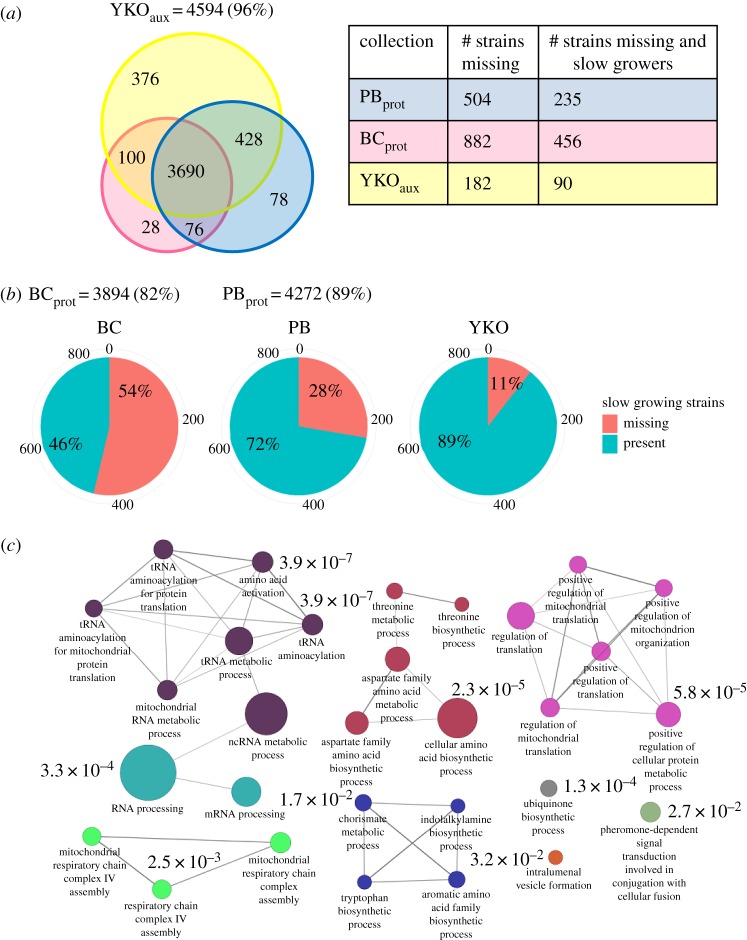
Genetic knockout strain make-up of the three deletion collections. (*a*) Venn diagram depicting deletion strains present in each of the three deletion collections in SC media (YKO_aux_, BC_prot_, PB_prot_) compared with the gene universe of strains present in at least one collection (4776). Table compares the number of strains missing (i.e. are below the background threshold) from each collection, and the subset of these strains that exhibit slow growth [33]. (*b*) Pie charts illustrating the proportion of 849 established slow-growing deletion strains [33] absent/present in each collection. Relative proportions of missing strains from each collection were comparable to a similar study [34]. (*c*) GO enrichment map of the 882 gene deletion strains absent in the BC_prot_ collection relative to the universe in (*a*). The two most highly enriched biological processes were mitochondrial RNA metabolism (*q* = 7.08 × 10^−5^) and amino acid biosynthetic processes (*q* = 2.35 × 10^−5^). Different node colours represent different GO biological processes; node size is proportional to the number of genes present in each GO term enrichment. The width of each edge is proportional to the degree of gene overlap between GO terms.

We constructed a robust metric for strain presence for the comparison of two prototrophic collections with the YKO_aux_ collection after generating independent pools of all deletion strains (Material and methods). Strain presence was quantified by microarray signal intensities following five generations of growth in synthetic complete (SC) media. The distribution of signal intensities was significantly different for each collection (Kolmogorov–Smirnov *p*-value < 0.05, electronic supplementary material, figure S2). To allow a fair comparison of the fitness profiles obtained, background thresholds were independently determined for each pool using a two-component Gaussian mixture model (Material and methods). The need to independently assess background thresholds was not unexpected; the distribution of fluorescence intensity for a given pool shifts towards lower values as the number of specifically bound probes decreases. Strains hybridizing below these thresholds were considered to be absent from their respective pool and were removed prior to any downstream analysis. A total of 4776 gene deletions were present in at least one pool and 77% (3690) of those were present in all three collections. The original YKO_aux_ collection represented the non-essential yeast genes most comprehensively with 96% (4594) of deletion strains, compared with 89% (4272) in the PB_prot_ collection and 82% (3894) in the BC_prot_ collection (figure 1*a*; electronic supplementary material, Additional File S1).

To highlight the differences between gene rosters of strains present or absent from each collection, pairwise combinations were evaluated for shared and unique functional enrichments. The most surprising difference was the 882 strains below the limit of detection missing from the BC_prot_ collection, 376 of which were also absent from the PB_prot_ collection. Of the 849 deletion strains annotated as required for optimal growth [33], 54% and 28% were missing strains from the BC_prot_ and PB_prot_ collections, respectively, compared with 11% for the YKO_aux_ collection (figure 1*b*). Overall, the 882 genes absent in BC_prot_ were significantly enriched for amino acid and nucleotide biosynthetic pathways (*q* = 2 × 10^−5^) in addition to all aspects of mitochondrial function (figure 1*c*; electronic supplementary material, Additional File S2). The absence of these strains was anticipated as the majority are slow or inviable in MM [33] and would, therefore, have been selected against during strain construction.

Of the 76 strains absent only in the YKO_aux_, no enrichment for biological processes was observed. Moreover, 60% (45) of these strains were never successfully constructed as diploids by the YKO deletion project. A small subset of strains was absent from the YKO_aux_ and BC_prot_ collections and explicitly required for mating (*COA1*, *GPA1*, *MSL1*, *SIR2*, *SIR3*, *SRV2*, *STE2*, *STE4*, *STE5*, *STE7*, *STE11*, *STE14*) [1,2,35,36], reflecting the inability of these strains to survive the mating step during construction. The relative proportions of strains present in SC media shown here were consistent with those following five generations of growth in rich media (YPD) (electronic supplementary material, figure S3). Barcode sequencing (Bar-seq) of select samples in this study was compared with published data from our laboratory and others (electronic supplementary material, figure S4) [29,30], providing an independent measure of strain presence which recapitulated the microarray data (electronic supplementary material, Additional Files S3 and S4).

### 2.2. Comparative fitness profiling

Following assessment of strain presence, we next characterized the phenotypic behaviour of each collection in competitive fitness assays performed in diverse stress conditions including (i) 13 nitrogen and nucleotide-limiting conditions, (ii) the DNA-damaging agent cisplatin and (iii) mitochondrial stress conditions: the oxidative phosphorylation uncoupling agents FCCP, CCCP and growth in obligate respiratory conditions (YPG) (table 2; electronic supplementary material, Additional File S3). For all assays, deletion pools were grown robotically and harvested after five generations of growth (Material and methods).

**Table 2.** Drug and media conditions assayed per deletion collection by microarray. *MM condition for YKO_aux_ was supplemented with histidine (20 mg l^−1^), leucine (30 mg l^−1^), methionine (20 mg l^−1^) and uracil (20 mg l^−1^). Additional Bar-seq experiments were done for the YKO_aux_ (ARG–, TRP–, LYS–, SC) and *MATa* (ARG–, TRP–, SC) with three and four replicates per condition, respectively (electronic supplementary material, Additional File S4).

control	condition	YKO_aux_	BC_prot_	PB_prot_
SC	control	3	3	3
SC	ADE–	3	3	3
SC	ARG–	3	3	3
SC	HIS–	0	3	3
SC	LEU–	0	3	3
SC	LYS–	3	2	3
SC	MET–	3	3	3
SC	SER–	3	2	3
SC	THR–	3	2	3
SC	TRP–	3	3	3
SC	URA–	0	3	3
SC	MM*	3	4	5
SC	MM + URA	0	1	1
YPD	control	11	14	12
YPD	cisplatin, DNA cross-linking agent	3	4	3
YPD	CCCP, protonophore inhibitor of oxidative phosphorylation	3	3	3
YPD	FCCP, protonophore inhibitor of oxidative phosphorylation	3	3	3
YPD	PYRQ, novel quinolone compound, PCID 16001701	4	4	4
YPD	YPG, obligate respiratory	2	4	4

#### 2.2.1. Nutrient-limiting conditions

**Figure 2.**
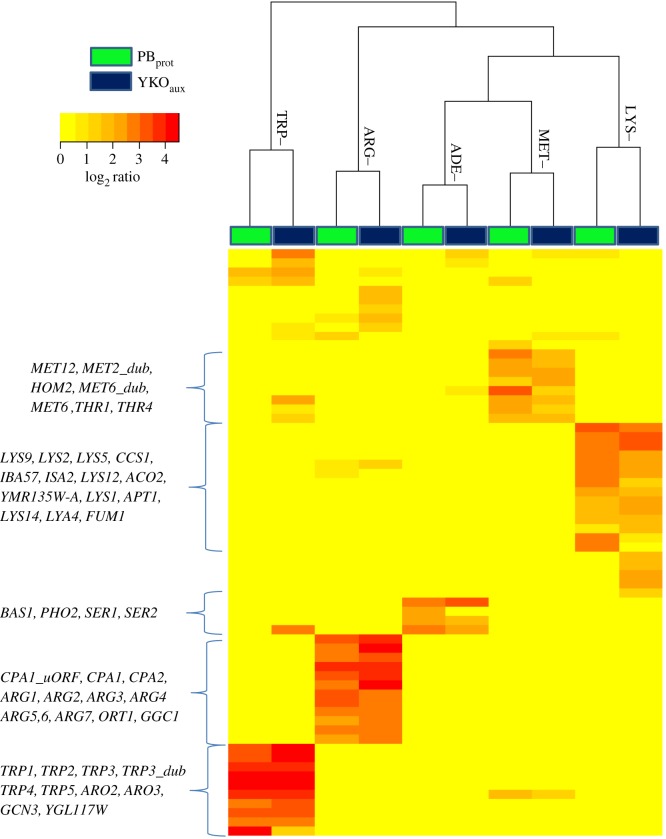
Comparative fitness profiling of the YKO_aux_ and PB_prot_ collections in synthetic dropout media. Hierarchical clustering of median fitness defect (FD) scores for deletion strains across three replicates observed for YKO_aux_ (blue) and PB_prot_ (green) in five synthetic dropout conditions (ADE–, ARG–, MET–, LYS–, TRP–) (log_2_ ratio ≥1 in at least one condition). The labelled clusters list the deletion strains with FDs in the corresponding dropout media.

**Figure 3.**
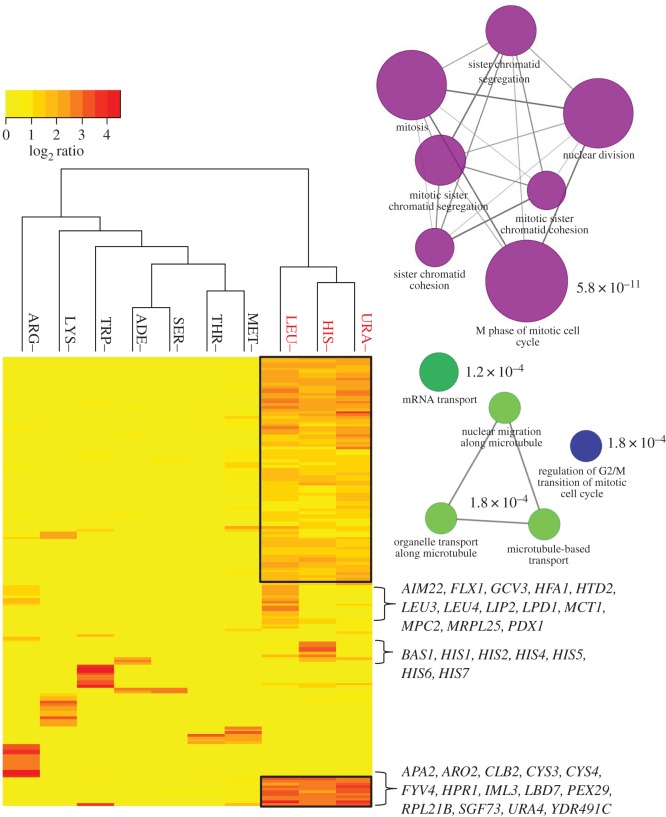
Characteristic PB_prot_ fitness signature observed in conditions requiring expression from the auxotrophy-complementing HLUM plasmid. Hierarchical clustering of median FD scores for deletion strains (log_2_ ratio ≥1 in at least one experiment) for the PB_prot_ collection in synthetic dropout media (ADE–, ARG–, HIS–, LEU–, LYS–, MET–, SER–, THR–, TRP–, URA–). Characteristic deletion strains belonging to the HLU (shared in HIS–, LEU– and URA– conditions) plasmid signature are boxed in black. Inset: GO biological process enrichment for the HLU signature. Node colours represent different GO biological processes; node size is proportional to the number of genes present in each GO term enrichment. Edge widths in the network represent the fraction of overlap between genes in related GO terms.

Fitness profiles readily identify all genes required in the corresponding biosynthetic pathways when assayed in conditions lacking a specific amino acid, purine or pyrimidine [37]. Both the YKO_aux_ and the PB_prot_ collections recapitulated the established biosynthetic pathways with only minor differences observed between collections (Pearson's correlation *r* = 0.91–0.96) in adenine (ADE–), arginine (ARG–), methionine (MET–), lysine (LYS–) and tryptophan (TRP–) dropout screens (figure 2; electronic supplementary material, figure S5*a–d*). The conditions that prohibit screening of the YKO_aux_ collection, including histidine (HIS–), leucine (LEU–) and uracil (URA–) dropout media, were of the greatest interest because of the paucity of functional annotations for these biosynthetic pathways. In these conditions, expression of the genes carried on the PB_prot_ ARS-CEN vector is explicitly required and fitness profiling of the PB_prot_ revealed a unique gene signature that described genes required for plasmid and mini-chromosome maintenance (figure 3). The fact that the histidine–leucine–uracil (HLU) fitness signature was not observed in MET– media despite requiring active expression of *MET17* from the ARS-CEN vector (figure 2; electronic supplementary material, figure S5) is consistent with methionine's role in the regulation of cell cycle progression. Insufficient levels of intracellular methionine (and its downstream product, cysteine) signal cell cycle arrest at G1/start [38–40] until a sufficient level of metabolites is reached to allow successful progression of the cell cycle. During methionine depletion, this cell cycle delay may alleviate the fitness defects (FDs) observed for HIS– LEU– and URA– in the HLU signature. If this interpretation is correct, we expect to observe a similar gene signature in any condition that impinges on histidine, leucine or uracil biosynthetic pathways.

The HLU fitness signature is defined by 73 core genes and revealed an enrichment for biological processes that involved a response to DNA replicative stress including: (i) nuclear division (*q* = 2.75 × 10^−12^), (ii) regulation of mitotic sister chromatid segregation (*q* = 2.54 × 10^−11^) and (iii) M-phase of mitotic cell cycle (*q* = 1.02 × 10^−12^) (figure 3, inset; electronic supplementary material, Additional File S5, https://goo.gl/AnUx9o). Many of these genes were originally identified in classic genetic screens for chromosome instability [41], including *CLB5, CSM3, CTF4*, *CTF19*, *ELG1, IML3, MCM16*, *MCM21, MRC1, NUP120* and *RTT109*.

**Figure 4.**
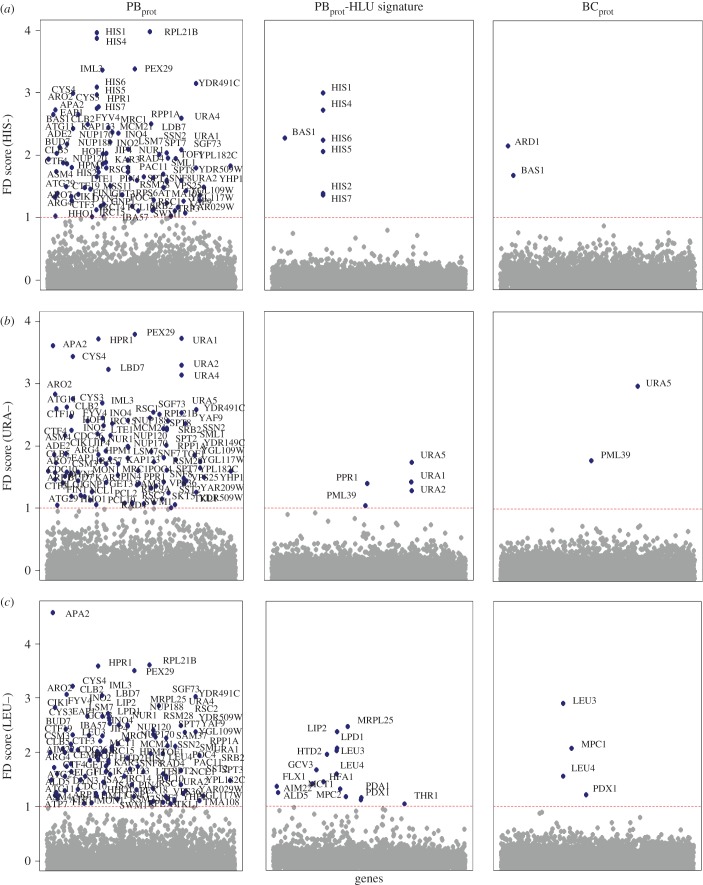
Fitness profiles of prototrophic collections in HIS–, LEU– and URA– dropout conditions. Fitness profiles for the PB_prot_ and BC_prot_ deletion collections in (*a*) HIS–, (*b*) URA– and (*c*) LEU– after five generations of growth. Left panel: FDs observed for the PB_prot_. Middle panel: PB_prot_ fitness profiles after correcting for the 73 genes in the HLU signature (Material and Methods), revealing histidine-, leucine- and uracil-specific effects. Right panel: fitness profiles observed for the BC_prot_ collection in the same condition. Red dashed line indicates the significance threshold of an FD score of 1.0.

To test if we could correct for this confounding effect and allow the identification of genes specifically sensitive in HIS–, LEU– and URA– screens, fitness scores were recalculated using the HLU gene signature as the reference condition (Material and methods). Following this data transformation, resulting fitness profiles clearly identified genes required in these biosynthetic pathways (figure 4*a–c*). The histidine profile revealed all genes required for biosynthesis, as well as the general amino acid control (GAAC)-regulated *BAS1* transcription factor required specifically in adenine- and histidine-limiting conditions (figure 4*a*). Similarly, the uracil profile identified *URA1*, *URA2* and *URA5* as required for uracil biosynthesis in addition to the *PPR1* transcriptional activator of the *de novo* pyrimidine biosynthesis pathway. As *URA4* was a member of the common HLU signature, it was not identified as specifically being required in the uracil dropout condition (figure 4*b*). The presence of these auxotrophic strains in the PB_prot_ collection was unexpected. We have subsequently learned that the construction of the PB_prot_ collection [9] involved the initial selection of transformants on *either* histidine *or* uracil single dropout conditions that was followed by passaging on MM (M. Ralser 2016, personal communication). The ultimate reason for the presence of auxotrophic strains in the PB_prot_ collection, therefore, remains to be determined.

Compared with *HIS3* and *URA3* auxotrophy, deletion of *LEU2* is considered more detrimental to cellular physiology, exhibiting slower growth rates [9] and a decreased rate of survival in starvation conditions [30,42]. In leucine dropout conditions, PB_prot_ FDs included *HTD2* from the mitochondrial fatty acid biosynthetic pathway (FASII), genes involved in protein lipoylation (*AIM22, GCV3*, *LIP2*), and *LPD1* and *PDX1* encoding the mitochondrial dihydrolipoyl dehydrogenase complex. Consistent with these results, the BC_prot_ leucine profile reported *PDX1*, as well as *MPC1,* a subunit of the mitochondrial pyruvate carrier (*MPC1/MPC2*), and the transcription regulators *LEU3* and *LEU4*. As such, the strains incurring FDs in both collections are deleted for genes that regulate beyond those explicitly required for leucine biosynthesis (figure 4*c*). These observations are of particular interest as leucine is thought to play a role in central metabolism, including iron–sulfur cluster biogenesis, mitochondrial genome maintenance, and regulation of acetyl-CoA between mitochondrial and cytoplasmic compartments [43]. The FASII pathway is thought to provide the octanoic acid required for biosynthesis of the cofactor lipoic acid (*AIM22, GCV3*, *LIP2*), which in turn is required by the mitochondrial pyruvate dehydrogenase complex (*LPD1, PDX1*), suggesting that the leucine biosynthetic pathway plays a substantial role in maintaining healthy mitochondrial function [33]. To date, however, the exact role of the FASII pathway has not been directly demonstrated and, therefore, the relationship between leucine metabolism and the FASII pathway, which cannot be evaluated in the YKO_aux_ collection due to leucine auxotrophy, serves to highlight an advantage of the prototrophic collections.

Because the majority of deletion strains required for amino acid and nucleotide biosynthesis are missing from the BC_prot_ collection, the resulting FDs were observed only in strains that are specifically compromised for growth in dropout, but not in MM (on which the prototrophic deletion strains were selected). Reported fitness scores included several strains deleted for genes in the GAAC system, including *ARO3*, *ARO4*, *GCN3* and *GCN20,* also observed in the YKO_aux_ and PB_prot_ collections (electronic supplementary material, figure S5). *GCN4,* however, was present only in the YKO_aux_ collection due to the requirement for this important transcriptional activator during the nutrient selection steps required for the construction of both prototrophic collections.

**Figure 5.**
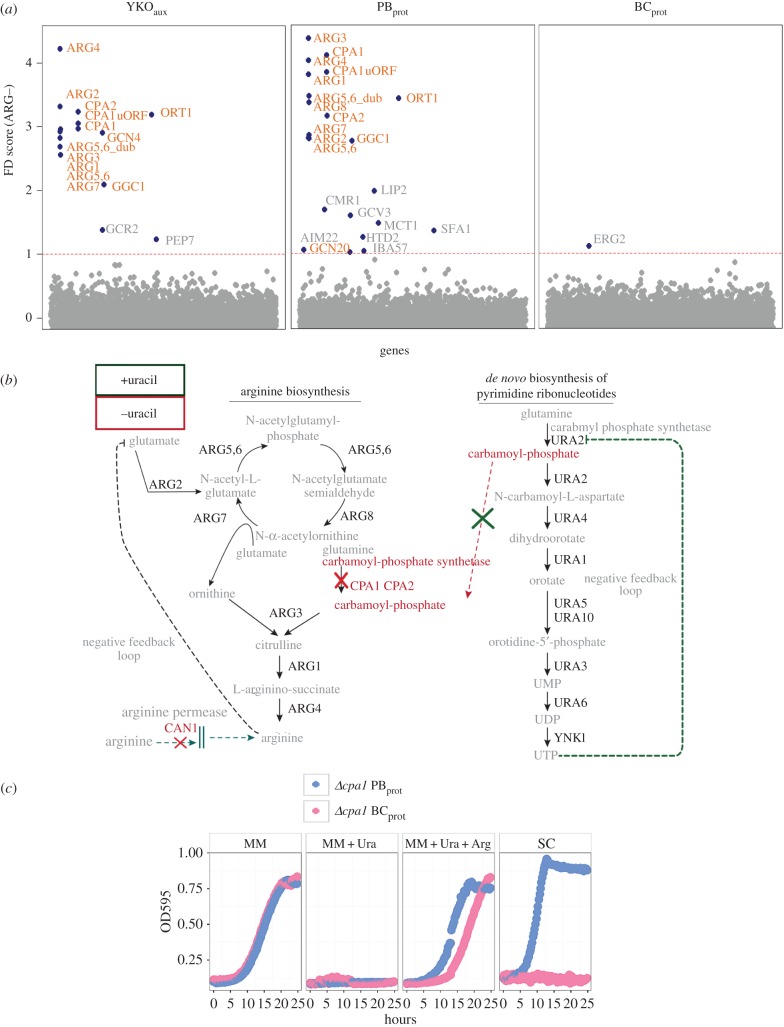
Fitness profiling reveals genotype-specific biology. (*a*) Fitness profiles in ARG– for the YKO_aux_, PB_prot_ and BC_prot _deletion collections. Orange labels: FDs in genes known to be required for arginine biosynthesis. Red dashed line indicates the FD significance threshold of 1.0. (*b*) Schematic depiction of the cross-talk between the arginine and pyrimidine biosynthetic pathways via the metabolite carbomyl phosphate. In the absence of arginine and uracil supplementation (red), the *cpa1Δ* strain grows normally by ‘borrowing’ carbamoyl phosphate produced by the pyrimidine biosynthetic pathway. If uracil is added (green), the pyrimidine pathway is repressed, and growth of the *cpa1Δ* strain is prohibited, phenocopying the synthetic lethal interaction of *ura2Δ* with *cpa1Δ*. (*c*) Testing of collection-specific phenotypes observed for the *cpa1Δ* strain by individual strain analysis. The PB_prot _and BC_prot _*cpa1Δ* strains grow normally in minimal media (MM), and neither grows in the presence of exogenous uracil (MM + Ura). Arginine amounts present in standard SC media (86 mg ml^−1^) is sufficient to rescue the PB_prot _*cpa1Δ* phenotype (SC), as is true for the YKO_aux _*cpa1Δ* strain (data not shown). By contrast, even arginine concentrations 150× higher than in SC (1.3 × 10^4^ mg ml^−1^, MM + Ura + Arg) are insufficient to fully rescue the BC_prot _*cpa1Δ* strain due to the deletion in *CAN1* (encoding the major arginine transporter) present in the BC_prot_ genetic background.

A unique BC_prot_ FD observed was for the strain deleted for *CPA1*, a gene required for arginine biosynthesis. The *cpa1Δ* BC_prot_ strain exhibited severe FDs in all conditions *except* in minimal and uracil dropout media (electronic supplementary material, figure S6). By contrast, the YKO_aux_ and PB_prot_ *cpa1Δ* strains were sensitive only in arginine dropout media (figure 5*a*). These results were observed using both the YKO_aux_ and the *MATa* collection (electronic supplementary material, figure S7). Because the *can1Δ* (encoding the major arginine transporter) in the genetic background of the BC_prot_ collection (table 1) is synthetically lethal with genes in the arginine pathway, we investigated how the *cpa1Δ* strain was able to survive selection on MM during strain construction and why it manifested such an unusual phenotype. An explanation was provided by a classical biochemical study [45] examining the regulation of pyrimidine biosynthesis and the two genes, *CPA1* and *URA2,* that encode carbamoyl-phosphate synthase (CPS) (figure 5*b*, –uracil). The activity of either is sufficient to supply the metabolic product carbomyl phosphate, required for both arginine and pyrimidine biosynthesis. In MM, *URA2* is expressed and active, allowing the *cpa1Δ* strain to grow normally. Addition of uracil to the media represses the *URA2* enzyme through negative feedback, and *cpa1Δ* is inviable due to the absence of any carbomyl phosphate synthetase activity (figure 5*b*, +uracil). Viability can be rescued by adding arginine to the media as evidenced by the PB_prot_ *cpa1Δ* (figure 5*c*). However, the BC_prot_ *cpa1Δ* strain is also deleted for *CAN1* (encoding the major arginine transporter), which prohibits rescue even in the presence of excess arginine (figure 5*c*). This *can1Δ cpa1Δ* negative genetic interaction therefore explains the FDs observed for the BC_prot_ *cpa1Δ* in all conditions where uracil is present. Interestingly, in a previous study, a BC_prot_-specific *cpa1Δ* FD (as well as the *cpa1Δ_uORF*) was observed when grown in rich media with dextrose (YPD) that was alleviated in rich media with galactose providing the sole carbon source (YPGal) (log ratio −3.5 and −2.9, respectively) [28], suggesting a depression of the pyrimidine pathway in line with the known requirement for UTP in YPGal metabolism. Taken together, the can1(deletion symbol)cpa1(deletion symbol) phenotypes described above are consistent with the need for an active pyrimidine biosynthesis pathway in order to rescue these strains' FDs (electronic supplementary material, figure S8).

#### 2.2.2. Drug and small-molecule stress conditions

**Figure 6.**
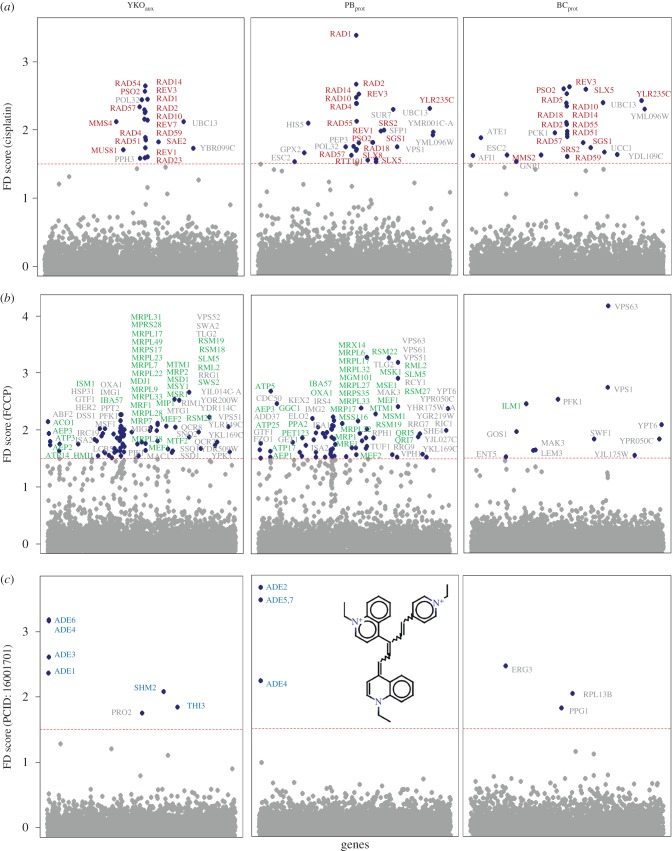
Comparative chemogenomic fitness profiling. Fitness profiles of the median FD scores across three replicates for the YKO_aux_, PB_prot_ and BC_prot_ deletion collections in (*a*) the DNA cross-linking agent cisplatin, (*b*) the proton ionophore FCCP and (*c*) a novel quinolone compound (PCID 16001701) [32]. Red, blue, green labels: FDs observed for strains deleted for genes annotated in DDR (DNA damage response), adenine biosynthesis and mitochondrial processes, respectively. Red dashed line indicates an FD score of 1.5.

Results from chemogenomic profiling of the DNA-cross-linking agent cisplatin for all three collections were consistent with established mechanisms and previous genome-wide fitness studies [46–48]. Strains exhibiting drug sensitivity in all three collections were significantly enriched for specific DNA damage response (DDR) processes that included, for example, nucleotide excision repair (NER) (*RAD1, RAD2, RAD4, RAD10, RAD14*), homologous recombination repair (HRR) (*RAD51, RAD55, RAD59*), post-replication repair (PRR) (*RAD5, RAD18*), translesion synthesis (TLS) (*REV1, REV3*) and *PSO2,* which is required for repair of cisplatin-induced inter-strand cross-links (figure 6*a*). Interestingly, *HIS5* appears with a significant FD in the PB_prot_, but not in the YKO_aux_ or BC_prot_ profiles (figure 6*a*). As there is cross-talk between the histidine and adenine pathways, it is possible that there is an impact on histidine biosynthesis during DDR. In addition, of the 36 genes involved in DNA damage with a significant FD score in at least one collection, approximately 60% (22) exhibit slow growth only in YPD but not in MM. Reflecting the ability of the PB_prot_ and BC_prot_ to maintain these strains during collection construction in MM, approximately 75% of these genes were present in those two collections, compared with approximately 25% in the YKO_aux_ collection.

In *S. cerevisiae,* approximately 1000 of all 6000 genes participate in mitochondrial processes and serve crucial, evolutionarily conserved cellular functions. We therefore focused on conditions that perturb mitochondrial function to compare deletion collections. Fitness profiles of the three collections in low doses of the mitochondrial membrane potential poisons CCCP and FCCP as well as growth in obligate respiratory media (YPG, where glycerol provides the carbon source) exhibited strong enrichment in both YKO_aux_ and PB_prot_ profiles for mitochondrial translation and respiration (*q* < 1 × 10^−17^ in all conditions). By contrast, the BC_prot_ fitness response was relatively sparse; no enrichment was observed in any of the mitochondrial stress conditions, reflecting the significant proportion of mitochondrial deletion strains missing in that collection (figure 6*b*).

Challenging the deletion collections with a compound of unknown mechanism provides an unbiased stress for comparing the three collections. The fitness signature of a cationic quinolone (PCID 16001701) previously screened by our laboratory [32,49] was highly correlated with adenine dropout fitness profiles (electronic supplementary material, figure S5*a*). We reproduced this gene signature in the YKO_aux_ which was supported by a similar profile in PB_prot_, identifying adenine and folic acid biosynthesis genes (*ADE1*, *ADE3*, *ADE4*, *ADE6*, *SHM2, THI3*) (figure 6*c*). The profiles from these two collections suggest the compound acts via a mechanism that requires adenine biosynthesis directly or indirectly. The BC_prot_ profile was uninformative with respect to the mechanism of action of this compound.

## 3. Discussion

Our comparative, genome-wide fitness survey of the original YKO_aux_ and two prototrophic versions of the collection across diverse environmental and stress conditions revealed several surprising findings relevant to applying these collections in gene function studies. First, while both the PB_prot_ and BC_prot_ satisfy the definition of prototrophy, ‘that a cell or organism has the same nutritional requirements as wild- type’, the benefits of prototrophy are offset by the cost of losing informative deletion strains. For example, the selection on MM during the construction of the prototrophic collections by definition prohibits future study in these basic nutrient conditions, as informative strains unable to grow will be selected against. Our study demonstrates that these required selection steps introduced both predictable and unexpected biases. Specifically, only approximately 25% of the 882 strains missing from the BC_prot_ collection were anticipated based on these selection steps. Of the remaining 670 strains, 57% (380) were also missing from the PB_prot_ collection, about half of which were identified as slow growers. To avoid misinterpretation in analysing fitness profiles, it is important to be aware of the biological processes associated with these missing strains. While the repair of the YKO auxotrophies by genetic complementation in PB_prot_ was more effective than backcrossing to a prototrophic strain (with respect to strain loss), it was not neutral. Phenotypic differences between episomal and integrated genetic complementation are well documented [50,51]. We therefore expect that, despite our ability to successfully correct for a well-defined HLU signature, unanticipated episomal effects are likely to occur that will escape detection. By contrast, though the BC_prot_ collection restored prototrophic markers to their native location (with the exception of the *HIS3* orthologue from *S. pombe* from a non-*HIS3* promoter), the *CAN1* and *LYP1* deletions present in the genetic background also introduced biases, as demonstrated for the *cpa1Δ can1Δ* synthetic lethal phenotype. These effects disrupt a key feature of competitive fitness assays—namely that the relative strain abundance in the starting pool is approximately equal. Nutritional selection steps skew this initial distribution, particularly when multiple strain passages are part of the construction methodology. As a result, the ability to detect FDs becomes more difficult, as reflected by the divergent background thresholds and lower signal intensity distributions of the prototrophic collections, compared with the YKO_aux_ collection (electronic supplementary material, figure S2).

The unexpected liabilities present in the prototrophic collections underscore that highly engineered versions of YKO_aux_ deletion collections are more constrained than generally assumed. Informative strains lost from the BC_prot_ collection (approx. 900 strains) share significant biological enrichment for genes involved in mitochondrial processes that compromise the ability to interrogate these processes (figure 1*c*). Consistent with this finding, we found that this set of strains was also absent in the *MATa* haploid SGA. The SGA collection serves as the starting point for the study of synthetic genetic interactions [52], yet approximately 1800 total strains are not detectable by barcode microarray hybridization signal (electronic supplementary material, figure S9), limiting the biological space surveyed by SGA and related deletion collections requiring sequential selective pinning assays. Nonetheless, the value of these and other collections and technologies in providing biological insight beyond the scope of the original YKO is indisputable.

The ability to perform such a precisely genetically controlled study on three genotypically distinct deletion collections in *S. cerevisiae* is not currently feasible in other systems. Our results therefore may provide insight into fundamental principles of genotype-by-environment relationships. For example, although the concept of robust genetic buffering is pervasive in the literature (primarily from the systematic study of digenic interactions), our results also suggest that condition-dependent cellular responses (i.e. phenotype) are greatly influenced by genotype.

The experimental design and assay constraints described here may help guide screens in other organisms and cell lines as they become tractable using CRISPR and other genome-editing techniques. Systematically benchmarking genomic libraries will be critical to establishing and maintaining the quality of functional and phenotypic gene annotations. Finally, we hope our study will serve to encourage and guide the design of future yeast deletion collections, most notably the need to move beyond derivative YKO_aux_ libraries to the de novo construction of a truly prototrophic collection.

## 4. Conclusion

This work underscores the degree to which systematic genetics and genomics has advanced our understanding of genotype–phenotype relationships. The resolution of comparative fitness profiling is highly sensitive, providing detailed biological insight and revealing methodological biases inherent in strain construction. Furthermore, this work demonstrates that despite differences in protocols, laboratories and experimental read-out, the results presented here can readily be extended to meta-analyses. We hope that these results encourage systematic comparative genomics of more divergent yeast collections such as those described for pseudo-filamentous or enological strains [53,54] and closely related human pathogens [55].

## 5. Material and methods

### 5.1. Yeast deletion strains and media preparation

The YKO_aux_ and *MATa* deletion collections are from the original stock centre of the *Saccharomyces* Genome Deletion Project [1], curated and maintained by Angela Chu at the Stanford Genome and Technology Center. The PB_prot_ [9] and BC_prot_ [25] deletion collections were kindly provided by the Ralser and Caudy laboratories. Synthetic complete and amino acid dropout media were purchased from Sunrise Science Products. The curators of Saccharomyces Genome Database (SGD) [7] provided the data that 76% of the phenotypic annotations (111 504 out of a total 146 128) were derived from the YKO (personal communication, March 2017).

### 5.2. Deletion pool construction

The diploid YKO_aux_, haploid *MATa*, BC_prot_ and PB_prot_ collections were pinned (S&P Robotics Inc., BM3- BC) from thawed glycerol stocks in 384-well or 96-well plates, respectively, onto rich YPD media (20 g l^−1^ bacto peptone, 10 g l^−1^ yeast extract, 20 g l^−1^ bacto agar and 20 g l^−1^ glucose), and recovered for 48–72 h at 30°C until colonies reached 2 mm in diameter. Plates were flooded with 12 ml liquid media and yeast cells were soaked and scraped off the plates. Resuspended cells from each plate were pooled in a sterile flask, and the final OD_600_ of the pool was adjusted to a final 50 OD_600_ ml^−1^. DMSO was added to the pool to a final concentration of 7% (v/v), mixed well, and the final pool aliquoted into individually capped PCR tubes and stored at −80°C.

### 5.3. Competitive fitness assays: synthetic media screens

Pooled deletion strains were diluted to starting OD_600_ of 0.0625 in 700 μl and were grown in duplicate wells on the same plate for five doublings in a Tecan Genios (Tecan Systems, Inc.) spectrophotometer at 30°C. Cells were manually harvested (synthetic media and drug screens) or automatically collected (YPG screens) using a Packard Multiprobe (PerkinElmer) liquid handler and stored at −20°C in a 48-well plate for no longer than 1 day. For the amino acid dropout experiments, each pooled collection was grown in SC medium or rich medium (YPD) as the control condition, and synthetic medium with an individual amino acid of interest dropped out as the experimental condition. For the YPG experiments, 3% glycerol was the experimental condition and YPD was the control condition.

### 5.4. Competitive fitness assays: chemical screens

Samples were subject to the same starting OD_600_ and doubling times as above. Screening concentrations for each compound (cisplatin (Toronto Research Chemicals), PCID 16001701 (ChemDiv), CCCP (Sigma), FCCP (Sigma)) were determined by analysing dose–response curves on wild-type auxotrophic BY4743 or prototrophic BY4743 transformed with p*HLUM* to determine the concentration that inhibits BY4743 growth by 15–20% (200 µM for cisplatin, 125 µM for PCID 16001701, 32 µM for CCCP and 6 µM for FCCP). YPD plus solvent (2% DMSO) was used as the controls with the exception of cisplatin (2% H_2_O).

Following growth, genomic DNA was extracted from cell pellets using the YeastStar Genomic DNA Kit (Zymo Research, catalogue #D2002) and quantified using the NanoDrop 2000 (Thermo Scientific). Uptag and downtag barcodes were amplified separately, pooled and hybridized to the Affymetrix TAG4 microarray (Genflex tag16K array) as previously described [27,32].

### 5.5. Array normalization and preprocessing

Each probe on the TAG4 barcode microarray (Genflex tag16K array, Affymetrix) is represented by five replicate features dispersed across the array that allow hybridization artefacts to be identified and corrected. Hybridization artefacts were removed using a previously described masking algorithm [27]. Independent sample sets were defined by collection and growth media (six sets in total, PB_prot_, BC_prot_ and YKO_aux_ in SC and YPD). To define background thresholds independently for each pool, we used a two-component Gaussian mixture model to fit the distribution of tags in the control arrays in each set (R v. 3.2.2, mixtools package, v. 1.0.4) [56,57]. The estimated components represent tags that successfully hybridized (present) and tags that did not hybridize (absent). We used the posterior distribution of the assignment of a tag to the present or absent component to select present tags for further analysis in non-control array data. To be called as present, all tags representing an ORF had to have a posterior value greater than 0.5 in all of the control replicates.

During the course of this study, we recognized that the homozygous BY4743 pools we used in this study were missing a subset of 143 strains due to a technical error that occurred during pool construction. These strains are part of the YKO v. 2.0 (http://www-sequence.stanford.edu/group/yeast_deletion_project/ykov2.html) that had already been added to the *MATa* versions of the collections available from commercial suppliers. Because our homozygous strain collection is derived from the original Stanford collection, these strains were omitted during shipment. In our study of the homozygous collection, the presence of these strains is supported by more than 3000 experiments [32]. This small subset of strains was used only for the purposes of the Venn diagram presented in figure 1*a*.

Next, tags designated as present upstream (uptags) and downstream (downtags) of the drug resistance cassette were normalized separately to their overall median across arrays within each of the six sets (the PB_prot_, BC_prot_ and YKO_aux_ collections in SC and YPD media). The uptag and downtag were collapsed into a single value by selecting the ‘best’ tag defined by the tag that exhibited the lowest coefficient of variation across the control replicates for each set. Biological replicates for each condition were performed in triplicate and batch corrected for technical variation using the ComBat function available in the R sva package (v. 3.14.0) [58].

### 5.6. Fitness defect scores

FD scores for each tag in each set were calculated by subtracting the log_2_ intensity of each individual tag in the treatment condition from the corresponding median in the control conditions. To estimate strains exhibiting significant FD scores, values from independent triplicate experiments were fit to a linear model; *q-*values (threshold *q* < 0.05) were obtained from the resulting *p-*values using the Benjamini—Hochberg method to adjust for multiple comparisons [59]. Pearson's correlations between the YKO_aux_ and PB_prot_ collections were calculated using deletion strains that had an FD score greater than one in at least one common nutrient-limiting condition. Fitness profiles for each condition (figures 2–6; electronic supplementary material, figures S1, S5, S7 and S8, Additional File S4) were summarized by the median value across triplicate FD scores. Similarly, the PB_prot_ HLU signature was defined by the median FD across the HIS–, URA– and LEU– replicates. To identify specific FDs in HIS–, URA– and LEU– dropout conditions, the PB_prot_ HLU common signature was subtracted from each of the HIS–, LEU– and URA– dropout conditions. To compare the overlap between FDs observed in Bar-seq versus those observed in the microarray analysis, only the experiments common to both were used (electronic supplementary material, Additional File S4).

### 5.7. GO enrichment

GO enrichment analysis was performed in Cytoscape (v. 3.3) [60] with the ClueGO plugin (v. 2.2.5) [61]. Enrichments for strains missing in the BC_prot_ collection (figure 1*c*) and deletion strains in the HLU signature (figure 3, inset) were compared with the gene universe (defined by the set of strains present in at least one of the three deletion collections). GO biological process terms with less than five genes or greater than 300 genes were excluded from the enrichment analysis. A right-sided hypergeometric test was used with a Bonferroni step-down correction and a minimum *p-*value of 0.0005 with a kappa score threshold of 0.4 [62]. Node sizes shown in the figures were proportional to the number of genes found in the gene set associated with the term.

### 5.8. Individual strain analysis

The BC_prot_ and PB_prot_ *cpa1Δ* strains were grown individually from a starting OD_600_ of 0.0625 to saturation in MM, SC, MM + uracil and MM + uracil + arginine (1.3×10^4^ mg ml^−1^) as shown in figure 5*c* or as described for pooled growth.

### 5.9. Library preparation

Bar-seq libraries were prepared using a custom two-step PCR approach using Phusion High-Fidelity DNA Polymerase (Thermo Fisher). First, uptags and downtags were separately amplified as described above for competitive fitness assays, but using primer pairs UP_F TCGTCGGCAGCGTCAGATGTGTATAAGAGACAGGATGTCCACGAGGTCTCT and UP_R GTCTCGTGGGCTCGGAGATGTGTATAAGAGACAGGTCGACCTGCAGCGTACG or DOWN_F TCGTCGGCAGCGTCAGATGTGTATAAGAGACAGGAAAACGAGCTCGAATTCATCG and DOWN_R GTCTCGTGGGCTCGGAGATGTGTATAAGAGACAGCGGTGTCGGTCTCGTAG. Uptag and downtag PCRs were then pooled in equal amounts and purified using the GeneJET PCR purification kit according to the manufacturer's instructions (Thermo Fisher). Second, purified barcodes were diluted 1 : 10 and 1 µl was used as template in the second PCR using Nextera XT index primers (Illumina), which contain individual barcodes as well as Illumina adapters. Cycling conditions were as follows: 98°C for 30 s; eight cycles of 98°C for 10 s, 55°C for 30 s, 72°C for 15 s; 72°C for 5 min. Libraries were then purified using Agencourt AMPure XP beads (Beckman Coulter) at a ratio of 3 : 5 beads to DNA, checked on Agilent High Sensitivity DNA chips for the Bioanalyzer (Agilent) and quantified using Quant-iT high sensitivity dsDNA Assay kit (Thermo Fisher). Pooled sequencing libraries were sequenced on a HiSeq 2500 (Illumina) in rapid run mode, generating paired or single-end 100 bp reads.

### 5.10. Bar-seq analysis

For the Bar-seq libraries sequenced with a paired-read protocol, the read mates were merged into single reads using BBMerge v. 8.82 from the BBTools/BBMap analysis suite (https://sourceforge.net/projects/bbmap/). Following that preliminary step, the same analysis procedure was then used on the reads originating from all the libraries, sequenced with paired or single-read protocols. Briefly, Bar-seq single sequence reads were first trimmed to 50 bases with Trimmomatic v. 0.33 [63] and then mapped to a yeast barcode database using the short-read aligner BWA v. 0.7.12 [64]. The BWA database was built using the barcode information from Pierce *et al.* [27] with the concatenation of barcode primer sequences at both ends of the barcodes specific for the uptags and downtags. Filtering of the aligned reads was performed with the SAMtools toolbox v. 1.2 [65], keeping only reads with mapping quality of 30 and above. Reads were counted for each library with the help of the BEDTools suite v. 2.24 [66] and a matrix of counts was created for the whole dataset with a custom-made Perl script for downstream statistical analysis. After filtering for tags that had greater than or equal to 50 counts across all control replicates, uptags and downtags for each strain were summed, normalized and analysed with the edgeR package v. 3.10.5 [67] as previously described [28].

## Supplementary Material

Genetic roster of each deletion collection by microarray intensity

## Supplementary Material

GO enrichment of strains absent in the BCprot

## Supplementary Material

Median log2ratios for comparative fitness profiling by microarray

## Supplementary Material

Average log2ratios for all conditions tested by Bar-seq

## Supplementary Material

GO enrichment of deletion strains present in the HLU signature

## Supplementary Material

Supplemental figure legends, additional files and references

## Supplementary Material

Figure S1

## Supplementary Material

Figure S2

## Supplementary Material

Figure S3

## Supplementary Material

Figure S4

## Supplementary Material

Figure S5

## Supplementary Material

Figure S6

## Supplementary Material

Figure S7

## Supplementary Material

Figure S8

## Supplementary Material

Figure S9

## Data accessibility

The datasets generated during the study will be available in the BioProject repository PRJNA338880 upon publication. The microarray data are deposited at GSE89761.
