# Supplementary material for: Comparative functional genomic screens of three yeast deletion collections reveal unexpected effects of genotype in response to diverse stress
Source: Open Biol. 2017 Jun 7;7(6):160330. doi: 10.1098/rsob.160330 (PMC5493772; doi:10.1098/rsob.160330)
Supplement: Supplemental figure legends, additional files and references [file rsob160330supp6.docx]

# Supplemental figure legends, additional files and references

Additional File 1: Genetic roster of each deletion collection by microarray intensity

Additional File 2: GO enrichment of strains absent in the BCprot

Additional File 3: Median log2 ratios for comparative fitness profiling by microarray

Additional File 4: Average log2 ratios for all conditions tested by Bar-seq

Additional File 5: GO enrichment of deletion strains present in the HLU signature

**Figure S1. Bar-seq of the YKO_aux_ and *MATa* collections commonly identify biosynthetic genes in synthetic dropout media.**

Fitness profiles of the median fitness defect scores across 3 replicates for the YKOaux and *MATa* collections in the amino acid dropout conditions (a) TRP- and (b) ARG- relative to the synthetic complete (SC) control. Both collections recapitulated the established biosynthetic pathways with only minor differences in the deletion strains with significant fitness defects. Red dashed line indicates a FD score of 1.5.

**Figure S2. Comparison of empirical cumulative density (ECDF) distribution for each collection.**

ECDF distributions calculated using the mean value from triplicate batch corrected SC controls for each collection. Kolmogorov-Smirnoff tests between each pair of deletion pools were used to estimate the significance of the signal intensity densities shown. All distributions were significantly different at a *P* value threshold of less than 0.05.

**Figure S3. Relative strain presence in YPD is consistent with that observed in SC media.**

Venn diagram of strain presence in YPD compared to SC for each collection, demonstrating > 97% agreement between the two conditions.

**Figure S4. Strain presence assessed by microarray is consistent, but less sensitive compared with deep barcode sequencing.**

Assessment of ~500 strains called absent by hybridization in the BCprot that were inconsistent with strains present by deep barcode sequencing (~10,000 counts/strain) (Bar-seq) from a previous study [1]. a) Distribution of array intensity for all genes. (b) Distribution of array intensities for the 500 strains called absent by array and present by Bar-seq. The lower intensity observed for these 500 strains did not pass the background array threshold, consistent with the established greater sensitivity of Bar-seq [[2](#_35nkun2)] compared to microarrays.

**Figure S5. Fitness profiles for all conditions.**

Collection-specific fitness profiles representing the average FD score of 4 control replicates (SC) to 3 treatment replicates for the YKOaux, PBprot, and BCprot deletion collections in synthetic dropout media (a) ADE-, (b) LYS- (c) MET- and (d) TRP-.

**Figure S6. Collection-specific phenotypic differences for the *cpa1Δ* strain.**

Fitness defects are shown for the *cpa1Δ*  strain across all three deletion collections for different synthetic media. The PBprot and YKOaux *cpa1Δ* strain exhibit fitness defects only in media containing uracil and lacking arginine. The BCprot *cpa1Δ* strain, in contrast, was only fit in media lacking uracil.

**Figure S7. Qualitative comparison of three haploid collections.**

Collection-specific fitness profiles representing the average fitness defect score of 4 control replicates (SC) to 3 treatment replicates in synthetic dropout media lacking arginine (ARG-) for the (a) *MATa* (Bar-seq), (b) PBprot and (c) BCprot collections (microarray). Strains with deletions for genes in the arginine biosynthetic pathway are seen for the *MATa* and PB_prot_, but not for the BC_prot_ profiles. Red dashed line indicates a FD score of 1.5.

**Figure S8. Rich media containing galactose alleviates fitness defects of the BCprot *cpa1Δ* strain.**

The fitness profile of the BCprot in YP-galactose [3] compared to YPD shows the expected sensitive strains including the GAL genes, while the *CPA1* and *CPA1_uORF* deletion strains exhibit resistance (as reflected in negative fitness defect scores).

**Figure S9. Shared enriched functions for strains missing from the BCprot, PBprot and SGA collections by microarray.**

The overlap in GO enrichment for missing strains in the BC_prot_, PB_prot_ and SGA collections reveals significant overlap for the BC_prot_ and the SGA, highlighting biology that cannot be interrogated with these collections. SGA hybridization intensities were from obtained from a previous SGA microarray experiment [4]. Node size and border corresponds to the number of genes in within the gene set and the significance of enrichment based on the BiNGO [5] *P* value (< 0.05) for the BC_prot_ (inner circle, red) and SGA (outer circle, grey), respectively. Edge size corresponds to the number of genes that overlap between the connected gene sets; green edges correspond to BC_prot_, blue edges to SGA, and purple edges to PB_prot_ collection.

# Supplemental References:

1. Gresham D, Boer VM, Caudy A, Ziv N, Brandt NJ, Storey JD, et al. System-level analysis of genes and functions affecting survival during nutrient starvation in Saccharomyces cerevisiae. Genetics. 2011;187:299-317.
2. Smith AM, Heisler LE, Mellor J, Kaper F, Thompson MJ, Chee M, et al. Quantitative phenotyping via deep barcode sequencing. Genome Res. 2009;19:1836-42.
3. Robinson DG, Chen W, Storey JD, Gresham D. Design and analysis of Bar-seq experiments. G3 (Bethesda). 2014;4:11-8.
4. Lissina E, Young B, Urbanus ML, Guan XL, Lowenson J, Hoon S, et al. A systems biology approach reveals the role of a novel methyltransferase in response to chemical stress and lipid homeostasis. PLoS Genet. 2011;7:e1002332.
5. Maere S, Heymans K, Kuiper M. BiNGO: a Cytoscape plugin to assess overrepresentation of gene ontology categories in biological networks. Bioinformatics. 2005;21:3448-9.
