# Supplementary figures and images for: Comparative functional genomic screens of three yeast deletion collections reveal unexpected effects of genotype in response to diverse stress
Source: Open Biol. 2017 Jun 7;7(6):160330. doi: 10.1098/rsob.160330 (PMC5493772; doi:10.1098/rsob.160330)

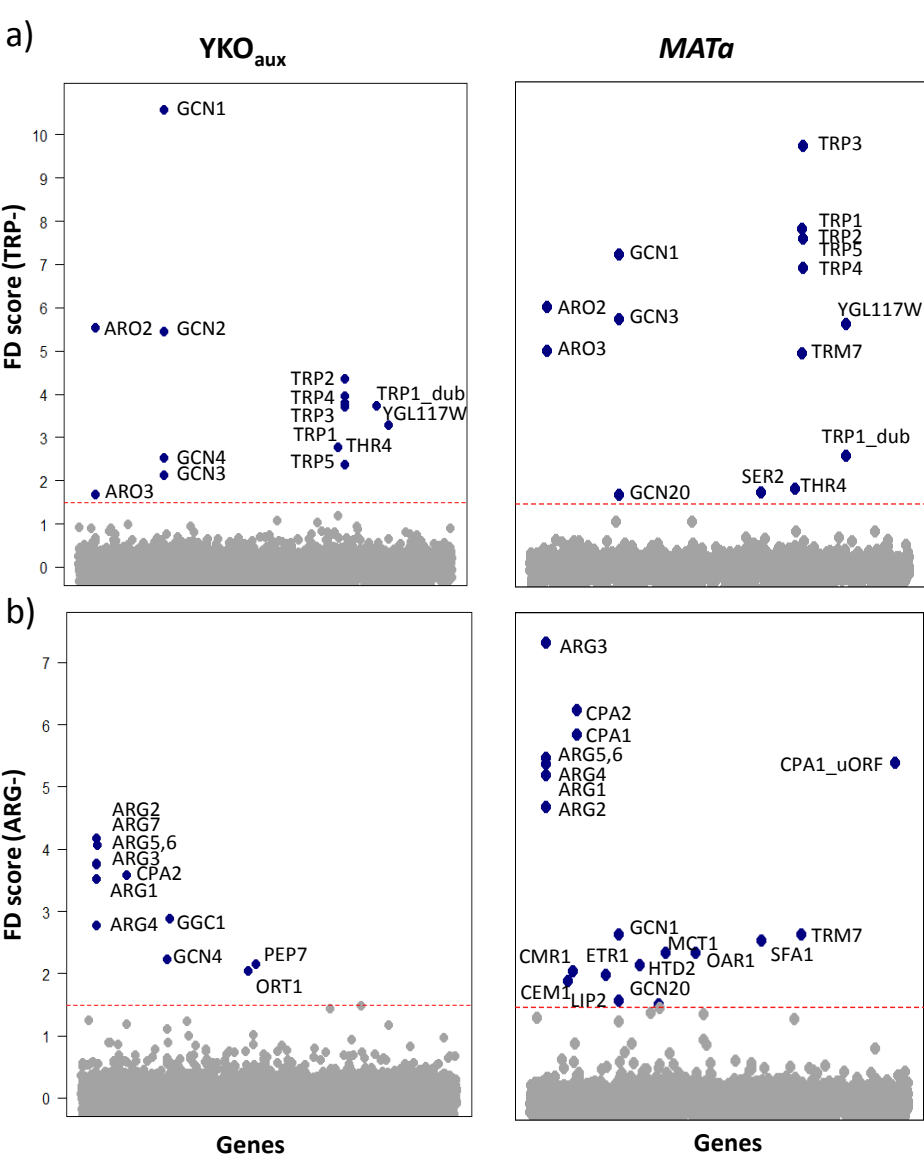

Supplement: Figure S1 [file rsob160330supp7.pdf]

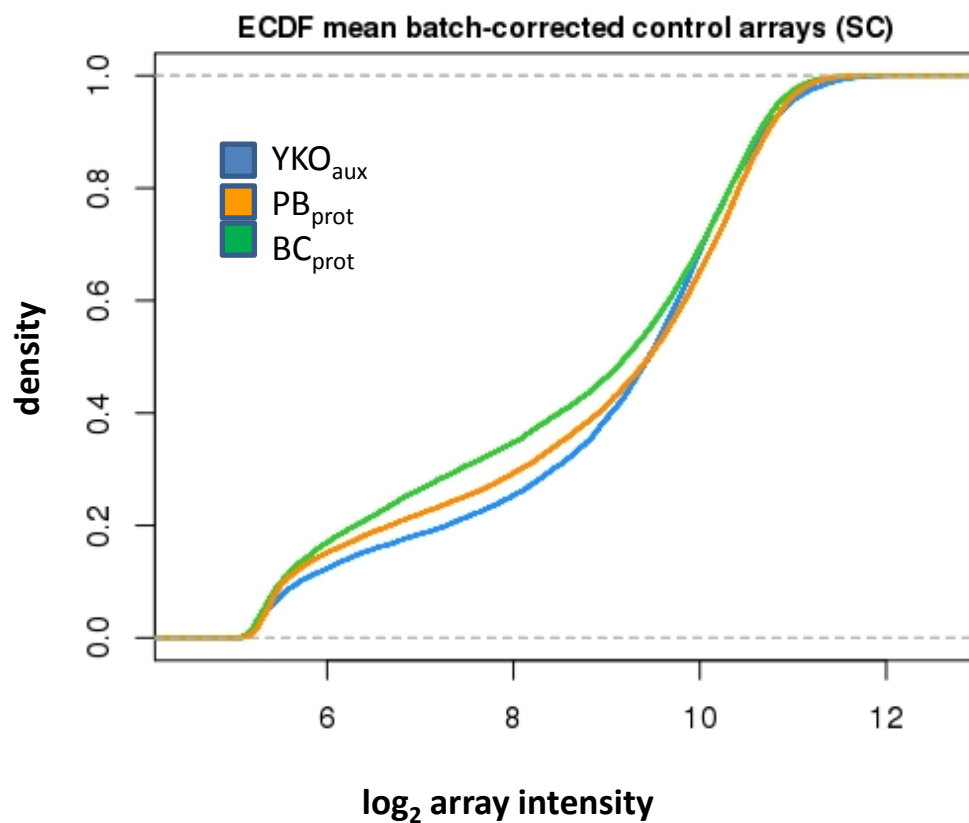

Supplemental Figure 2

Supplement: Figure S2 [file rsob160330supp8.pdf]

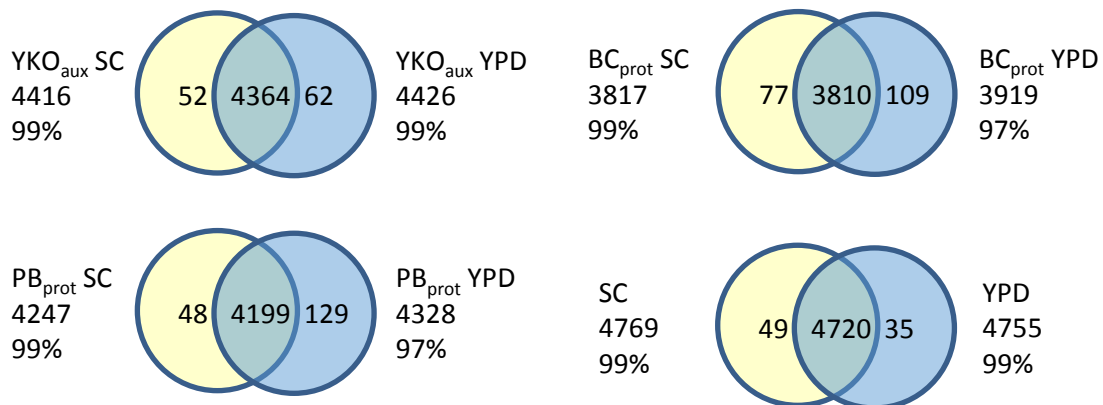

Supplement: Figure S3 [file rsob160330supp9.pdf]

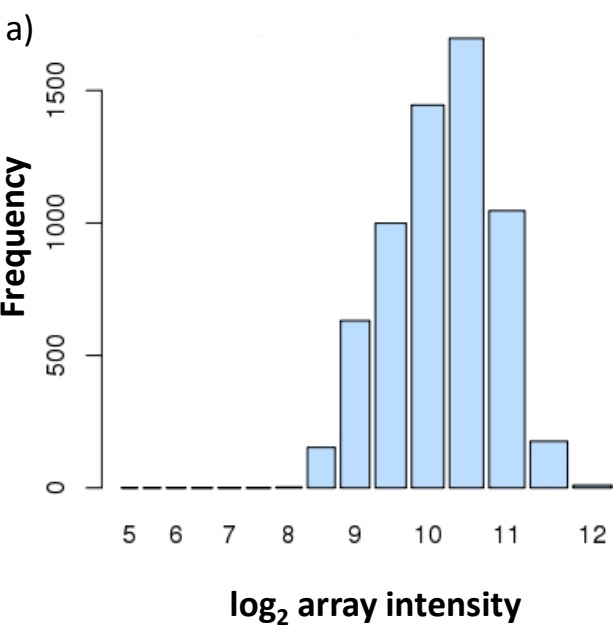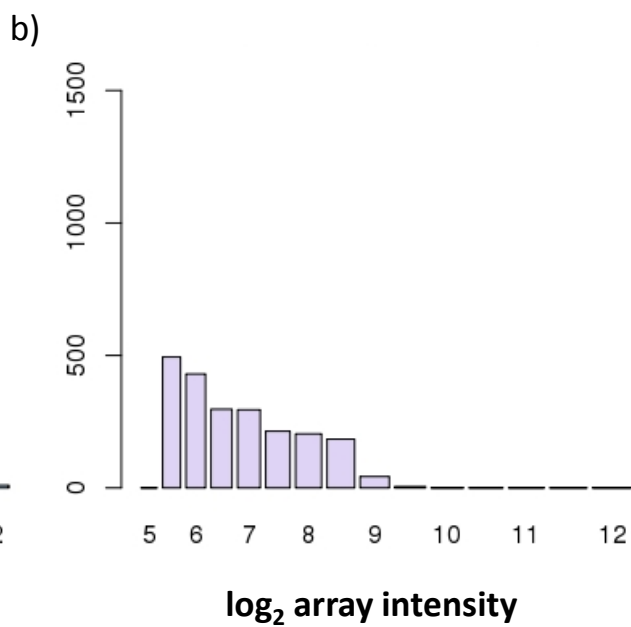

Supplement: Figure S4 [file rsob160330supp10.pdf]

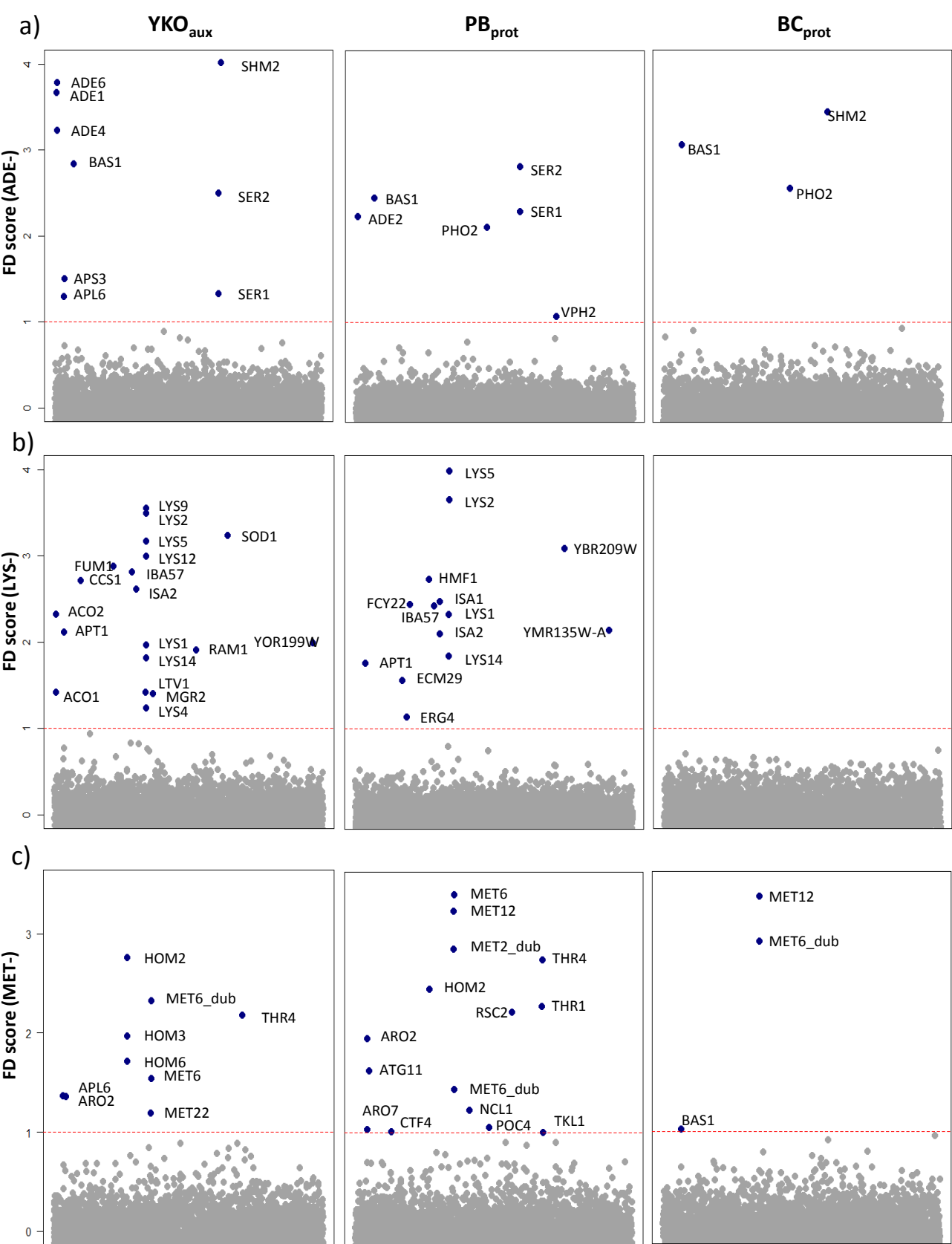

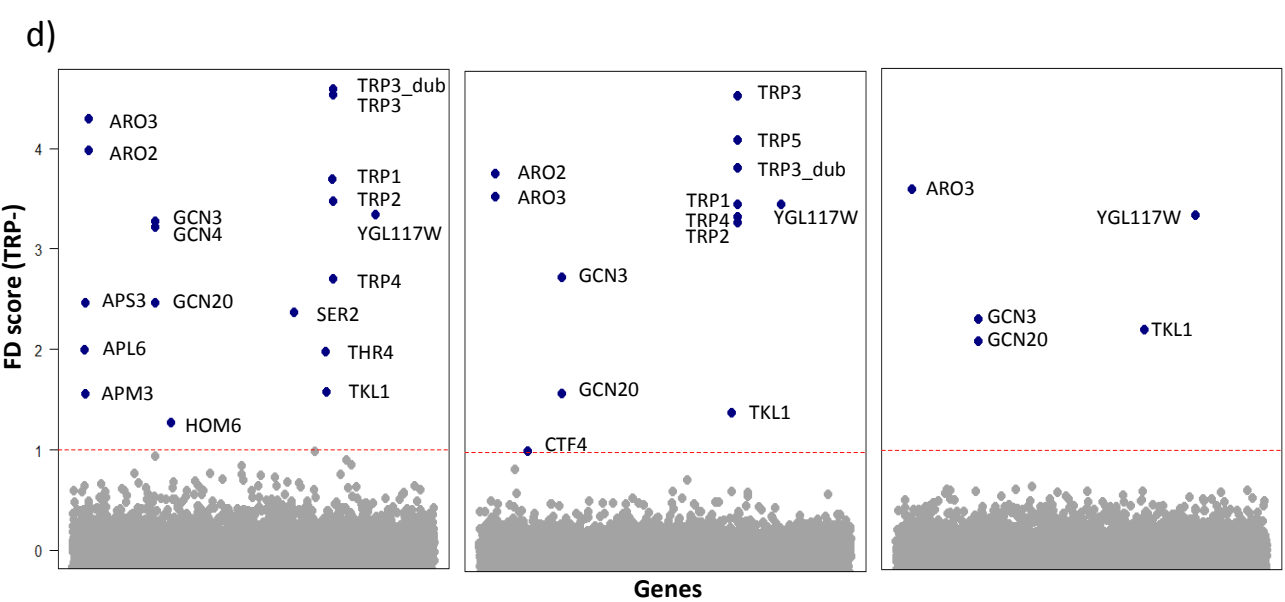

Supplemental Figure 5

Supplement: Figure S5 [file rsob160330supp11.pdf]

# Collection-specific phenotypes of the *cpa1* $\Delta$ strain

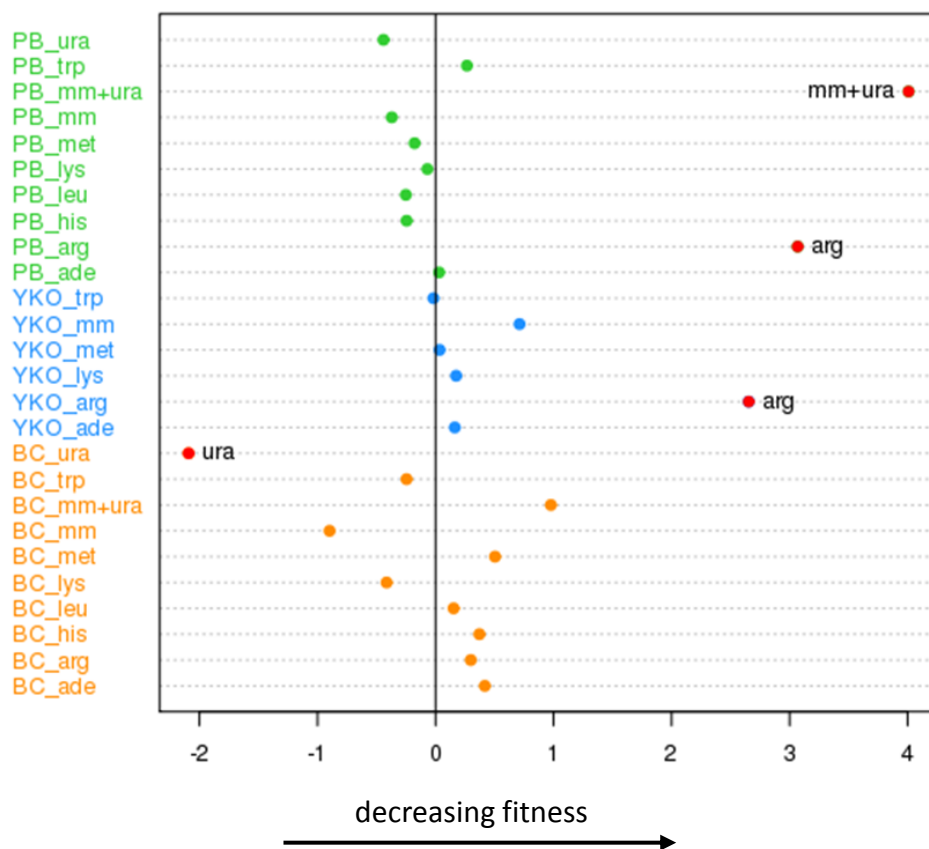

Supplemental Figure 6

Supplement: Figure S6 [file rsob160330supp12.pdf]

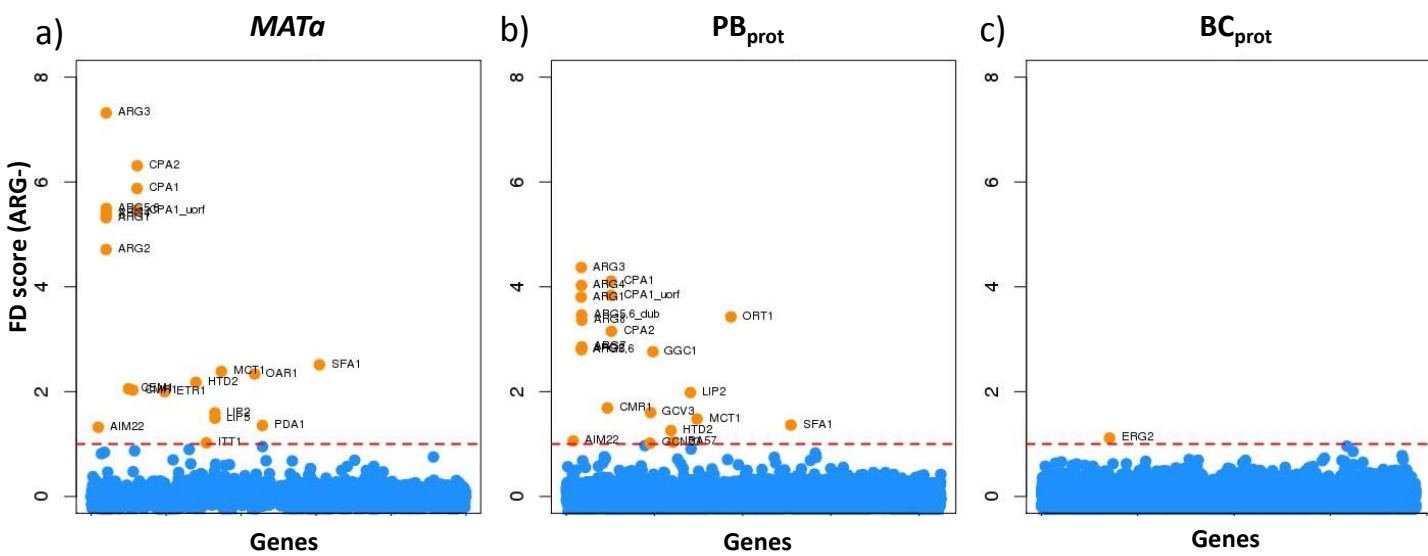

Supplement: Figure S7 [file rsob160330supp13.pdf]

BC<sub>prot</sub>

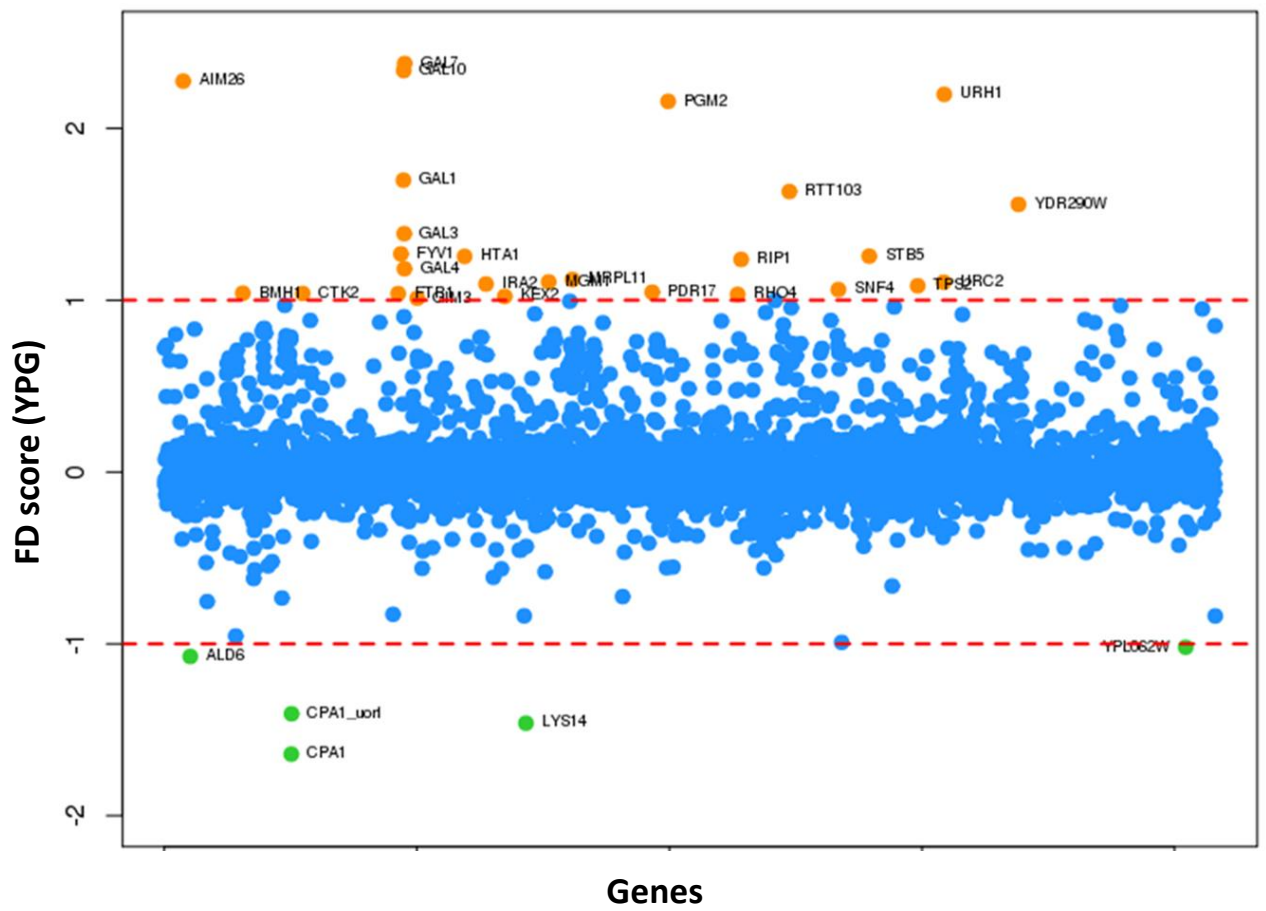

Supplement: Figure S8 [file rsob160330supp14.pdf]

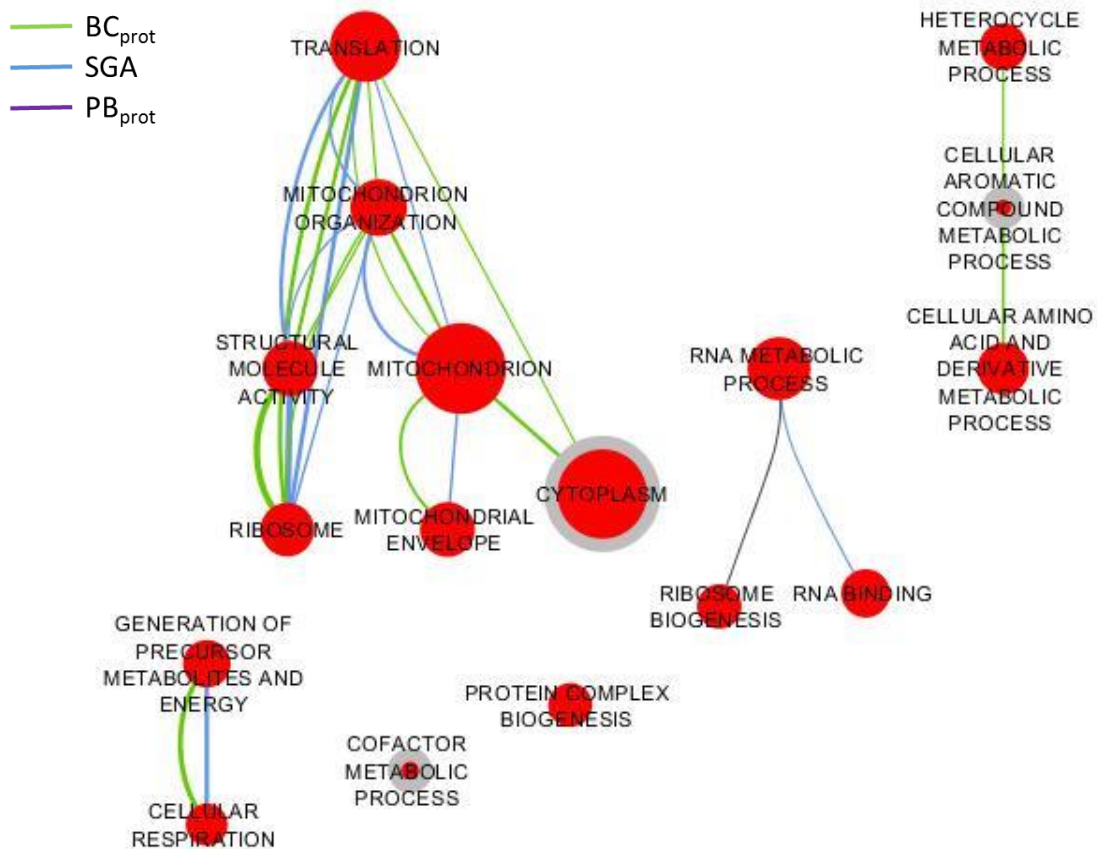

Supplement: Figure S9 [file rsob160330supp15.pdf]
